# Outcomes for 2 Children after Peripartum Acquisition of Zika Virus Infection, French Polynesia, 2013–2014

**DOI:** 10.3201/eid2308.170198

**Published:** 2017-08

**Authors:** Marianne Besnard, Timothée Dub, Patrick Gérardin

**Affiliations:** Centre Hospitalier de Polynésie Française, Pirae, Tahiti (M. Besnard); Institut Pasteur, Paris, France (T. Dub);; Centre Hospitalier Universitaire, Saint Pierre, Réunion (P. Gérardin**)**

**Keywords:** Zika virus, peripartum infection, neonatal hepatitis, French Polynesia, Tahiti, Reunion, viruses, outcomes

## Abstract

Congenital Zika virus infection is associated with severe brain anomalies and impaired function. To determine outcomes, we followed 2 affected children for ≈30 months. For 1 who was symptomatic at birth, transient hepatitis developed. However, neurodevelopment for both children was age appropriate.

Zika virus, a flavivirus, is a teratogenic and neurotropic infectious pathogen (*1*). Zika virus infection during pregnancy causes congenital microcephaly and severe brain anomalies (*2*). In the newly recognized congenital Zika syndrome, infection is also associated with partially collapsed skull, retinal damage, congenital contractures, early-onset hypertonia, and signs of extrapyramidal involvement; irrespective of a clear pathomechanism, infection is also associated with intrauterine growth restriction and low birth weight (*1*). Developmental outcomes for children born with congenital Zika virus infection have been reported for infants with severe brain anomalies as consequences of early prenatal exposure (*3*,*4*) and include postnatal slowing of head circumference growth and impaired function. 

After the first large-scale Zika outbreak in French Polynesia, October 2013–April 2014 (*5*), 2 cases of peripartum Zika virus infection in full-term neonates were reported (*6*). We report the follow-up and developmental outcomes through ≈30 months of age for these 2 children. We evaluated cognition by using the Child Development Assessment Scale (CDAS), a screening test suitable for children 0–5 years of age (Technical Appendix).

Case-patient 1 was born at 38 weeks’ gestation; his weight, size, and neurologic status were within reference ranges for gestational age. His mother manifested a rash, suggestive of Zika virus infection, on day 2 after delivery. Reverse transcription PCRs for Zika virus were positive in blood and saliva from the mother (day 2) and neonate (day 3) and in breast milk on day 2. The neonate was breastfed for 2 months. He remained asymptomatic, and his neurologic development followed a typical course. At 32 months of age, CDAS scores indicated a need to monitor motor development but overall did not indicate neurocognitive problems.

Case-patient 2 was also born at 38 weeks’ gestation but was small for gestational age (weight 1,925 g; height 42 cm; head circumference 32 cm). Signs of Zika virus infection (rash) appeared in the mother on day 3 and in the neonate on day 4. Reverse transcription PCRs for Zika virus of blood and urine were positive for the mother (day 1) and the neonate (days 4 and 7) and in breast milk on day 8. On day 2, laboratory testing of blood from the neonate indicated thrombocytopenia (65.0 × 10^9^ thrombocytes/L), leukopenia (4.6 × 10^9^ cells/L), cytolysis, and cholestasis (Table); the cholestasis resolved 4 months later. Ultrasonograms of the liver were unremarkable, and albumin levels and hemostasis remained within reference ranges. Breastfeeding was maintained for 6 months. At 30 months of age, the child’s growth remained within −2 SD for weight (10,725 g) and head circumference (47 cm) and –1.5 SD for height (86 cm). CDAS scores indicated no developmental neurocognitive problems.

**Table:** Follow-up of liver function test results associated with perinatal Zika virus infection in case-patient 2, French Polynesia, 2014*

Date (postnatal day)	AST, U/L†	ALT, U/L‡	GGT, U/L§	Bilirubin, total/conjugate, mg/L¶
Feb 4 (2)	**84**	11	**201**	**158**
Feb 6 (4)	38	12	**297**	**145**
Feb 10 (8)	**52**	18	**457**	**128/21**
Apr 8 (57)	**348**	**150**	**281**	**66/54**
Apr 16 (65)	**239**	**139**	**312**	**50/42**
Apr 30 (79)	**117**	**86**	**239**	10
May 13 (92)	**119**	**104**	**164**	8
Jun 17 (120)	**57**	**51**	**40**	2
Oct 25 (250)	38	**47**	27	2

*Boldface indicates values out of reference range. ALT, alanine aminotransferase; AST, aspartate aminotransferase; GGT, gamma-glutamyl transferase.  †Reference range 15–40 U/L. ‡Reference range 10–40 U/L. §Reference range 2–34 U/L. ¶Reference range <14/<3 mg/L.

Follow-up of these 2 case-patients showed that peripartum Zika virus infection, the exposure situation of mother-to-child transmission of Zika virus during gestation (when the mother is viremic during childbirth), was associated with neither marked illness at birth nor neurodevelopmental deficits by 30 months of age. However, assessment of late-onset cognitive or sensory deficits requires longer follow-up. Transmission is assumed unlikely to occur through breastfeeding; Zika virus isolated from milk is not replicative (*6*). For the 2 case-patients reported here, transmission by contact with the vaginal secretions seems unlikely; viral shedding in these secretions is scarce and weak (*7*).

Prolonged subclinical hepatitis in case-patient 2, such as that observed with congenital cytomegalovirus infection, resolved after 4 months. To the best of our knowledge, liver pathogenesis in living neonates with Zika virus infection has not been reported but is common with other arboviral (e.g., dengue virus) infections. This milder pattern of peripartum Zika virus infection differs from the usually severe neonatal dengue virus infection (severe thrombocytopenia) and peripartum chikungunya virus infection (postnatal encephalopathy).

Our findings, along with findings of mild brain lesions (e.g., subependymal cysts and lenticulo-striate vasculitis) after late intrauterine exposure to Zika virus (*8*), need to be replicated on larger populations before it can be suggested that placental and blood–brain barriers may be effective late in gestation, after fetal maturation. Studies on placental barrier function have produced discordant results (*9**,**10*). The primary human trophoblast cells of full-term placentae have been shown to be refractory to historic strains of Zika virus from Uganda (MR766) and Cambodia (FSS13025). However, a contemporary strain of Zika virus from Puerto Rico (PRVABC59) was able to infect human placental macrophages and mature primary human trophoblast cells (*9*). In addition, primary human trophoblast cells in a non–Zika virus–endemic population were permissive to a strain of Zika virus from Colombia (FLR) (*10*).

Taken together, uncertainty about the mode of transmission and discrepancies in epidemiologic study findings make it imperative to aggregate data to enable comparisons of the risk for transmission as a function of exposure during gestation. More accurate risk estimates should soon be possible thanks to efforts (meta-analyses of individual participant data of existing cohorts) of the international community, which should enable harmonized follow-up and evaluation of developmental outcomes for children exposed to Zika virus.

Technical Appendix Example of the Child Development Assessment Scale for case-patient 2.
