# Supplementary material for: Outcomes for 2 Children after Peripartum Acquisition of Zika Virus Infection, French Polynesia, 2013–2014
Source: Emerg Infect Dis. 2017 Aug;23(8):1421–3. doi: 10.3201/eid2308.170198 (PMC5547815; doi:10.3201/eid2308.170198)
Supplement: Technical Appendix — Example of the Child Development Assessment Scale for case-patient 2. [file 17-0198-Techapp-s1.pdf]

Article DOI: https://doi.org/10.3201/eid2308.170198

Outcomes for 2 Children after Peripartum Acquisition of Zika Virus Infection, French Polynesia, 2013–2014

Technical Appendix

The Child Development Assessment Scale

The Child Development Assessment Scale (CDAS) is a neurodevelopmental scale existing, both in French and in English (http://www.ged-cdas.ca). Developed in Québec by the Centre de Liaison sur l’Intervention et la Prévention Psychosociale (CLIPP), it is used to assess neurodevelopmental skills on three distinct dimensions: cognitive/language; motor and social-emotional. The fact that this scale strongly correlates with the Bayley Scales of Infant and Toddler Development®, Third Edition (Bayley-III®), a widely used neurodevelopmental scale, made it one of the most practical assessment tool in this setting (http://www.ged-cdas.ca/file_download/13/Standardization+study+CDAS.pdf). Before performing the evaluation, the examinator must enter the date of birth and gestational age (if the assessment is performed before 2 years old) of the assessed child, to decide which age-range specific questionnaire is to be used and allow automated corrections according to the score obtained (this latter depends on child’s corrected age). Each dimension is assessed separately and child’s score is classified in three categories: adequate development (or comfort zone), the gray zone corresponding to a domain that must be monitored, and the problematic development category, also known as referral zone. Case 1’s assessment at 32 months old was strictly adequate in both the cognitive/language and social emotional dimensions, while motor development remained in the gray area. As far as case 2 was concerned, when assessed at 30 months old, his results were adequate in all three categories, hence strictly normal.

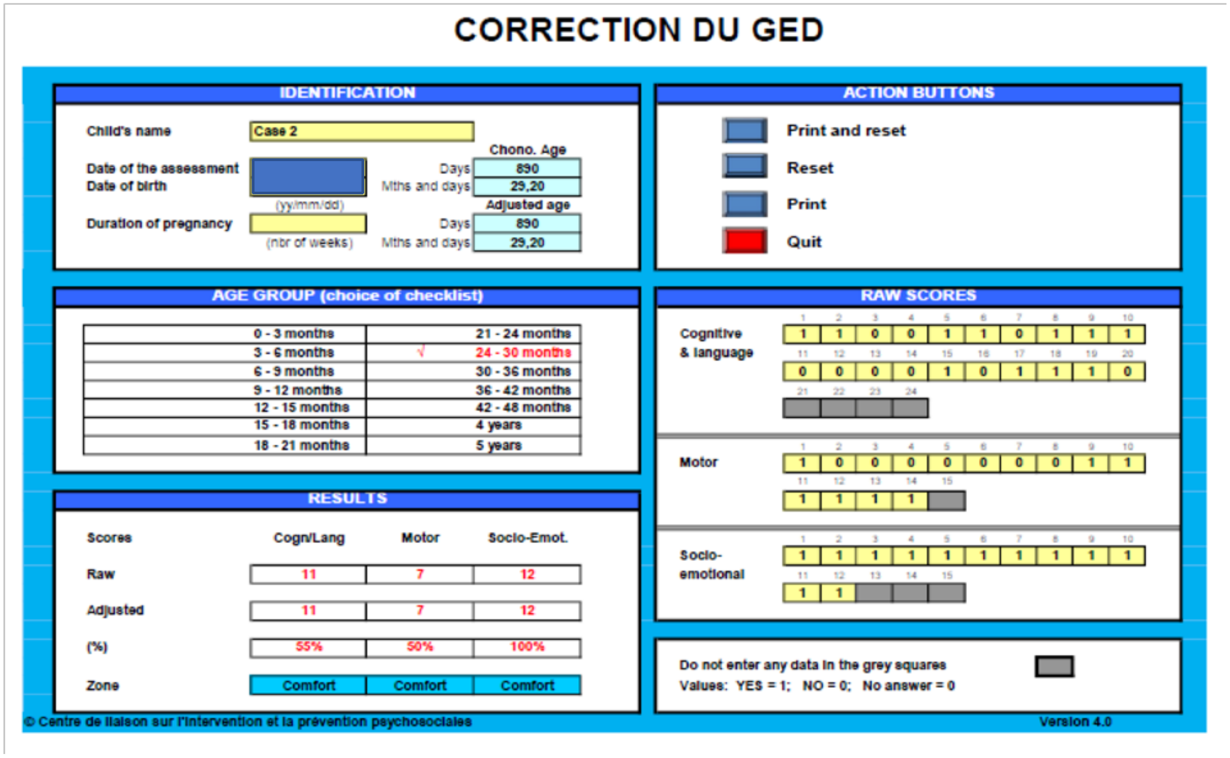

Technical Appendix Figure. Example of the Child Development Assessment Scale (GED) for case-patient 2. GED, Grille d'Evaluation du Développement.
